# Electric Field Control of Spin–Orbit Torque Magnetization Switching in a Spin–Orbit Ferromagnet Single Layer

**DOI:** 10.1002/advs.202301540

**Published:** 2023-06-17

**Authors:** Miao Jiang, Hirokatsu Asahara, Shinobu Ohya, Masaaki Tanaka

**Affiliations:** ^1^ School of Materials Science and Engineering Beijing Institute of Technology Zhongguancun South Street No.5, Haidian Beijing 100081 China; ^2^ Department of Electrical Engineering and Information Systems The University of Tokyo 7‐3‐1 Hongo, Bunkyo‐ku Tokyo 113‐8656 Japan; ^3^ Center for Spintronics Research Network (CSRN) Graduate School of Engineering The University of Tokyo 7‐3‐1 Hongo, Bunkyo‐ku Tokyo 113‐8656 Japan

**Keywords:** electric field control of magnetism, magnetization switching, single layer, spin–orbit ferromagnet, spin–orbit torque

## Abstract

To achieve a desirable magnitude of spin–orbit torque (SOT) for magnetization switching and realize multifunctional spin logic and memory devices utilizing SOT, controlling the SOT manipulation is vitally important. In conventional SOT bilayer systems, researchers have tried to control the magnetization switching behavior via interfacial oxidization, modulation of spin–orbit effective field, and effective spin Hall angle; however, the switching efficiency is limited by the interface quality. A current‐induced effective magnetic field in a single layer of a ferromagnet with strong spin–orbit interactions, the so‐called spin–orbit ferromagnet, can be utilized to induce SOT. In spin–orbit ferromagnet systems, electric field application has the potential for manipulating the spin–orbit interactions via carrier concentration modulation. In this work, it is demonstrated that SOT magnetization switching can be successfully controlled via an external electric field using a (Ga, Mn)As single layer. By applying a gate voltage, the switching current density can be solidly and reversibly manipulated with a large ratio of 14.5%, which is ascribed to the successful modulation of the interfacial electric field. The findings of this work help further the understanding of the magnetization switching mechanism and advance the development of gate‐controlled SOT devices.

## Graphical abstract

A promising technique is offered for efficient control of full spin–orbit torque magnetization switching in a spin–orbit ferromagnet single layer via an external electric field. This work helps understand the mechanism of magnetization switching from the viewpoint of the effective field, which paves the way toward combining novel high‐efficiency and low‐power spintronics memory devices and conventional field‐effect semiconductor technologies.

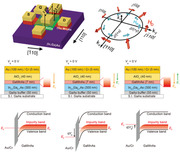

## 1 Introduction

Spin–orbit torque (SOT) has been proposed as a promising candidate to control the magnetization of ferromagnetic materials, showing great potential for the realization of spintronic memory devices with better stability, higher scalability, faster information processing speed, and lower energy consumption. To further enhance the SOT magnetization switching efficiency, researchers have explored various effective methods. First, the most generally used approach is to find materials with a large spin Hall angle *θ*_SH_ that can generate a large spin current, which can be utilized to effectively decrease the critical switching current density *J*_c_.^[^ ^1^, ^2^, ^3^, ^4^, ^5^ ^]^ Those materials with large *θ*_SH_ are utilized in bilayer systems, where the spin current generated in a nonmagnetic layer is injected into the adjacent ferromagnetic layer and exerts a torque to switch the magnetization. In bilayer systems, the SOT magnetization switching can be manipulated by controlling the interfacial oxidization,^[^ ^6^, ^7^ ^]^ modulating the spin–orbit interaction and the effective spin Hall angle.^[^ ^8^, ^9^, ^10^, ^11^ ^]^ However, the spin injection is limited by the interface quality, which hinders further decreases in *J*_c_. To overcome this limitation, a new class of materials called spin–orbit ferromagnets, including ferromagnetic alloy, ferromagnetic topological insulators and the ferromagnetic semiconductor Mn‐doped GaAs ((Ga, Mn)As), have recently been found to be very promising for highly efficient magnetization switching due to the strong spin–orbit‐induced effective magnetic field, the intrinsic bulk inversion asymmetry and the appropriate saturated magnetization.^[^ ^12^, ^13^, ^14^, ^15^ ^]^ Especially in (Ga, Mn)As, an extremely low *J*_c_ (4.6 × 10^4^ A cm^−2^) has been demonstrated by suppressing the contribution of the field‐like term with a current‐induced magnetic field.^[^ ^16^ ^]^ In a ferromagnetic alloy FePt single layer, a field‐free magnetization switching was achieved and a platform was introduced to engineer large SOTs for lower‐power spintronics devices by a composition gradient, while the switching ratio is limited and the *J*_c_ still needs to be decreased.^[^ ^17^, ^18^, ^19^ ^]^ In ferromagnetic topological insulators, electric field application has been demonstrated to control *J*_c_ via modulation of the density and type of surface carriers.^[^ ^15^, ^20^, ^21^ ^]^ However, the switching process is still improvable in terms of the switching hysteresis and its completeness.^[^ ^15^ ^]^ To achieve a desirable magnitude and realize multifunctional SOT spin logic and memory devices with a simple structure and high efficiency, manipulating the SOT in an appropriate spin–orbit ferromagnet single layer is strongly required. The single‐crystalline ferromagnetic semiconductor (Ga, Mn)As is one of the best material systems for this purpose, where the Dresselhaus and Rashba effective fields (*H*_D_ and *H*_R_) induced by the spin–orbit interactions contribute to an in‐plane spin component that can exert a strong torque on the magnetization.^[^ ^13^ ^]^ In addition to the conventional Dresselhaus and Rashba spin–orbit terms, there is also a contribution to the spin–orbit interaction resulting from the interface inversion asymmetry.^[^ ^22^ ^]^ Because the interfacial spin–orbit fields are demonstrated to be strongly influenced by the internal electric field,^[^ ^10^, ^11^ ^]^ external electric field application is expected to achieve effective manipulation of the SOT in (Ga, Mn)As.

**Figure 1:**
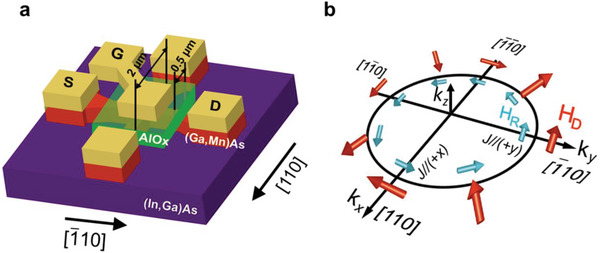
a) Schematic illustration of the SOT device structure. The channel width of the crossbar is 0.5 µm, and the channel length is 2 µm. The yellow parts are the capping layers on the electrodes consisting of Au (100 nm)/Cr (5 nm), which also work as heat sinks. The current *J* flows in the (Ga_0.94_, Mn_0.06_)As layer with perpendicular magnetization from the source electrode (S) to the drain electrode (D), and the Hall resistance *R*_H_ is obtained by measuring the Hall voltage *V*_H_ along the [110] direction orthogonal to the S–D direction. An electric field is applied by the gate voltage *V*_g_. b) Dresselhaus‐like (red) and Rashba‐like (light blue) effective magnetic fields (*H*_D_ and *H*_R_, respectively) for hole momenta along different crystallographic directions in the tensile‐strained (Ga_0.94_, Mn_0.06_)As thin film. (*k_x_*, *k_y_*, and *k_z_*) is the wave vector of holes.

In this Article, we demonstrate that the SOT magnetization switching behavior in a ferromagnetic (Ga, Mn)As single layer can be manipulated by applying an electric field using a solid gate electrode, as shown in **Figure** **1**a. By changing the sign and magnitude of the gate voltage *V*_g_, the interfacial electric field and hole concentration of (Ga, Mn)As are manipulated, enhancing or suppressing the spin–orbit interactions. Because the spin–orbit interactions couple the spin of a hole with its momentum and generate *H*_D_ and *H*_R_ (Figure 1b),^[^ ^23^, ^24^, ^25^, ^26^ ^]^ *H*_D_ and *H*_R_ are very sensitive to the applied electric field. By applying a positive *V*_g_, we show that the enhancement of *H*_D_ and *H*_R_ contributes to an increase in the SOT switching efficiency, decreasing *J*_c_. Our findings provide a promising method for efficient modulation of SOT switching by applying an electric field to a single spin–orbit ferromagnetic layer.

## 2 Magnetization Reversal in the Au/Cr/AlO*_x_*/(Ga, Mn)As System

**Figure 2:**
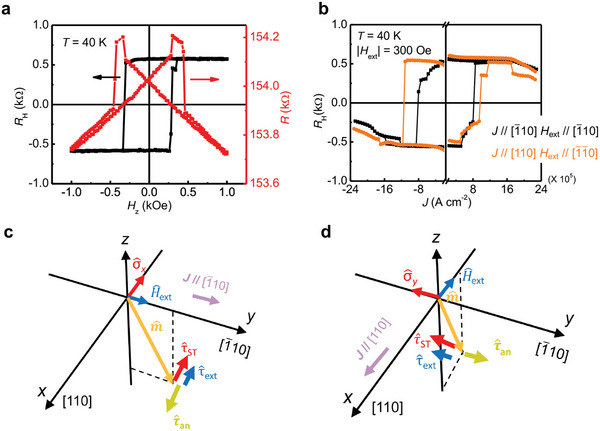
a) Perpendicular magnetic field *H*_z_ dependence of the Hall resistance *R*_H_ and resistance *R* of (Ga_0.94_, Mn_0.06_)As at 40 K. b) Field‐assisted SOT switching with *J* // [110] and [110] in a 7 nm thick (Ga_0.94_, Mn_0.06_)As thin film at 40 K, where the magnitude of *H*_ext_ is 300 Oe. c,d) Illustrations of the torques exerted by the spin component (${\hat{\tau }}_{\mathrm{ST}}$), the external field (${\hat{\tau }}_{\mathrm{ext}}$), and the anisotropy field (${\hat{\tau }}_{\mathrm{an}}$) with *J* > 0 for *J* // [110], *H*_ext_ // [110] c) and *J* // [110], *H*_ext_ // [110] d). $\hat{m}$ is in the *y–z* plane c) and the *x*–*z* plane d).

The sample structure examined in this study is (Ga_0.94_, Mn_0.06_)As (7 nm)/In_0.3_Ga_0.7_As (500 nm)/GaAs (50 nm) grown on a GaAs (001) substrate by molecular beam epitaxy (MBE). Due to the 500 nm thick lattice‐relaxed insulating In_0.3_Ga_0.7_As layer, a tensile strain is applied to the (Ga_0.94_, Mn_0.06_)As thin film, inducing perpendicular magnetic anisotropy (PMA). As shown in Figure 1a, the film was patterned into a crossbar (red parts in Figure 1a) with a size of 0.5 µm (width) × 2 µm (length) by electron beam lithography for transport measurements. The Au (100 nm)/Cr (5 nm) electrodes (yellow parts in Figure 1a), which also work as heat sinks, were deposited with electron beam evaporation. For the solid gate electrode, 40 nm thick AlO*_x_* was deposited by atomic layer deposition at 150 °C as a dielectric layer (see Figure S1 in the Supporting Information) Through characterization of the anomalous Hall effect (AHE), the PMA is confirmed by measuring the Hall resistance *R*_H_ with sweeping of a magnetic field *H_z_* applied perpendicular to the film plane. In **Figure** **2**a, the *R*_H_–*H_z_* curve (see the black curve) shows an obvious square‐like character, where *R*_H_ varies between approximately ±0.6 kΩ. The coercivity *H*_c_ is 300 Oe, as shown in the square‐like *R*_H_–*H_z_* curve, and we see an obvious anisotropic magnetoresistance (AMR) effect (see the red curve in Figure 2a), where the resistance *R* reaches the maximum value when the magnetization starts to be reversed by *H_z_*.

In addition to a magnetic field, a current *J* can also be utilized to switch the magnetization. As shown in Figure 2b, with the help of a small external magnetic field *H*_ext_ = 300 Oe for deterministic magnetization reversal, *R*_H_ can be changed between ±0.6 kΩ by sweeping *J* along the in‐plane direction, which is consistent with the AHE result in Figure 2a; this result indicates that the magnetization can be fully reversed between the +*z* and −*z* directions with a rotation angle of 180° by *J*. For *J* // [110] (see the black curve in Figure 2b and the illustration of torques in Figure 2c), the spin component $\hat{\sigma }$ *_x_* induced by the effective field is along the [110] direction, as shown in Figure 1b, which exerts an anti‐damping torque ${\hat{\tau }}_{\mathrm{ext}}$ on the magnetic moment. ${\hat{\tau }}_{\mathrm{ext}}$ is proportional to $\hat{m}\times {\hat{\sigma }}_{x}\times \hat{m}$, and the direction of ${\hat{\tau }}_{\mathrm{ST}}$is the same as that of $\hat{\sigma }$ *_x_*. Here, $\hat{m}\times {\hat{\sigma }}_{x}\times \hat{m}$ represents the unit magnetization vector. With the assistance of the torque ${\hat{\tau }}_{\mathrm{ext}}$ induced by *H*_ext_, i.e., ${\hat{\tau }}_{\mathrm{ext}}\;=\;-\hat{m}\;\times \;{\hat{H}}_{\mathrm{ext}}$ (here, ${\hat{H}}_{\mathrm{ext}}$ represents the vector of *H*_ext_), ${\hat{\tau }}_{\mathrm{ST}}$ overcomes the torque ${\hat{\tau }}_{\mathrm{an}}$ induced by the perpendicular anisotropy field *H*_an_, i.e., ${\hat{\tau }}_{\mathrm{an}}\;=\;-\hat{m}\;\times \;{\hat{H}}_{\mathrm{an}}$ (here, ${\hat{H}}_{\mathrm{an}}$ represents the vector of *H*_an_), and reverses the magnetic moment when *J* increases. In the case of *J* // [110] (see the orange curve in Figure 2b and the illustration of torques in Figure 2d), the induced spin component $\hat{\sigma }$ *_y_* points in the $\left[1\bar{1}0\right]$ direction, which is parallel to ${\hat{\tau }}_{\mathrm{ext}}$ and opposite to ${\hat{\tau }}_{\mathrm{an}}$. Therefore, with increasing *J*, ${\hat{\tau }}_{\mathrm{ST}}$ is enhanced, and magnetization reversal occurs. Here, we note that a small hysteresis window appears when *J* is between 12 × 10^5^ and 17 × 10^5^ A cm^−2^ along the $\left[1\bar{1}0\right]$ direction (see the black curve in Figure 2b), which might result from slight phase separation of the (Ga_0.94_, Mn_0.06_)As layer.

In the magnetization switching behavior shown in Figure 2b, *J*_c_ is 8.2 × 10^5^ A cm^−2^ at 40 K for *J* // [110]. By changing *J* toward the [110] direction, *J*_c_ is found to be increased to 10.5 × 10^5^ A cm^−2^. This occurs because *H*_R_ is induced in the Au/Cr/AlO*_x_*/(Ga, Mn)As system by the breaking of the structure inversion symmetry with the 40 nm thick AlO*_x_* layer and the Au/Cr electrode. As shown in Figure 1b, under *J* // [110], *H*_R_ is parallel to *H*_D_, which enhances the total effective field *H*_eff_ along the in‐plane direction, where *H*_eff_ = *H*_D_ + *H*_R_, and makes the spin component exert a strong SOT on the magnetization. In contrast, under *J* // [110], the direction of *H*_R_ is opposite to that of *H*_D_, which suppresses *H*_eff_ (*H*_eff_ = *H*_D_ − *H*_R_) and weakens the SOT. Therefore, the switching process is hindered by *H*_R_ when *J* flows along the [110] direction, resulting in an increase in *J*_c_. The aforementioned results indicate that the direction of the induced spin component is always along *H*_D_ (see Figure 1b). Hence, we can conclude that *H*_D_ is dominant during the SOT switching in the Au/Cr/AlO*_x_*/(Ga, Mn)As system and that *H*_R_ can assist the switching process for *J* // [110] but hinders it for *J* // [110].

## 3 Manipulation of SOT Magnetization Switching via a Gate Electric Field

**Figure 3:**
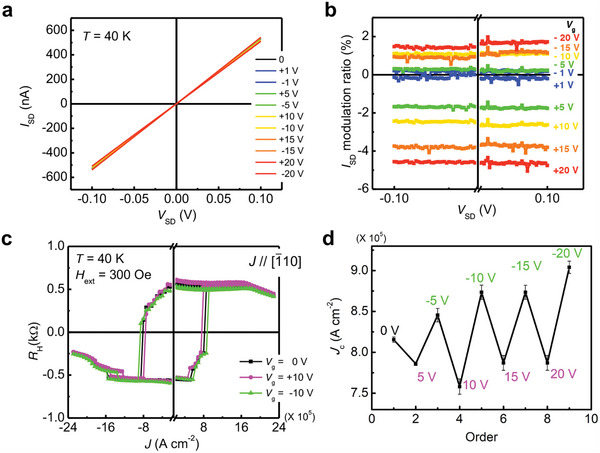
a) *I*_SD_–*V*_SD_ characteristics. b) *I*_SD_ modulation ratio with the application of various gate voltages *V*_g_ of 0, ±1, ±5, ±10, ±15, and ± 20 V at 40 K. *V*_SD_ ranging from +0.1 to −0.1 V was applied with a step of 0.001 V. c) Field‐assisted SOT switching by applying *V*_g_ of 0 and ±10 V. d) Plot of *J*_c_ obtained with various *V*_g_ in a 7 nm thick (Ga_0.94_, Mn_0.06_)As thin film at 40 K. Here, *J* // [110] and *H*_ext_ = 300 Oe. In this measurement, we changed *V*_g_ in the order of 0 V → +5 V → −5 V → +10 V → −10 V → +15 V → −15 V → +20 V → −20 V.

To achieve manipulation of the SOT magnetization switching with an electric field, we applied a gate voltage *V*_g_ to the gate electrode shown in Figure 1a. First, we measured the electrical characteristics of the crossbar device, where the source–drain current *I*_SD_ was measured as a function of the source–drain voltage *V*_SD_ at 40 K by applying various *V*_g_ of 0, ±1, ±5, ±10, ±15, and ± 20 V, as shown in **Figure** **3**a. Here, *V*_SD_ ranging from +0.1 to −0.1 V was applied with a step of 0.001 V. The *I*_SD_–*V*_SD_ curves in Figure 3a show an obvious ohmic character, meaning that ohmic contacts are formed in the crossbars. The contacts remained in good condition and were not destroyed during the measurements. By applying *V*_g_, we found that *I*_SD_ was varied, indicating that *I*_SD_ can be manipulated by *V*_g_. To more clearly understand the change in *I*_SD_ when applying different *V*_g_, *I*_SD_ modulation ratios at various *V*_g_ are plotted in Figure 3b based on the results shown in Figure 3a. Here, the modulation ratio is defined by ∆*I*_SD_/*I*_SD_ (*V*_g_ = 0) × 100%, where ∆*I*_SD_ is the *I*_SD_ modulation defined by ∆*I*_SD_ = *I*_SD_ (*V*_g_) − *I*_SD_ (*V*_g_ = 0). In Figure 3b, an obvious *I*_SD_ change that is modulated by *V*_g_ occurs. For *V*_g_ < 0, the *I*_SD_ modulation ratio is positive, indicating that the negative *V*_g_ increases the hole concentration in the (Ga_0.94_, Mn_0.06_)As layer. In contrast, for *V*_g_ > 0, the *I*_SD_ modulation ratio is negative, resulting from the decrease in the hole concentration in the (Ga_0.94_, Mn_0.06_)As thin film.

Next, SOT magnetization switching was induced by sweeping *J* along the [110] direction with the application of a *V*_g_ of ±10 V, as shown in Figure 3c. From the results, the application of the positive *V*_g_ = +10 V decreases *J*_c_ from 8.2 × 10^5^ to 7.6 × 10^5^ A cm^−2^, which means that the SOT switching efficiency is enhanced for *V*_g_ > 0. Meanwhile, for *V*_g_ = −10 V, *J*_c_ increases to 8.7 × 10^5^ A cm^−2^, indicating that the negative *V*_g_ hinders the switching process to some extent. Hence, by applying the electric field (*V*_g_ = ±10 V), *J*_c_ can be efficiently modulated by ≈14.5% [=(8.7 − 7.6)/7.6 × 100%]. Figure 3d shows the manipulation of the SOT switching (*J* // [110]) at different *V*_g_ of ±5, ±10, ±15, and ± 20 V at 40 K (see Figure S3 in the Supporting Information for details), from which we can conclude that a positive *V*_g_ decreases *J*_c_ and that a negative *V*_g_ increases *J*_c_. These results indicate that the magnetization switching behavior is solidly reversibly modulated via the electric field. Here, we note that the modulation of the *J*_c_ is not linearly correlated with the application of *V*_g_, which may be attributed to the extremely thin depletion layer and a limited modulation ratio of the practical interfacial electric field under *V*_g_ smaller than 20 V. To further enhance the modulation ratio under larger *V*_g_, further studies are needed.

## 4 Mechanism of the Electric Field Control of the SOT Magnetization Switching

**Figure 4:**
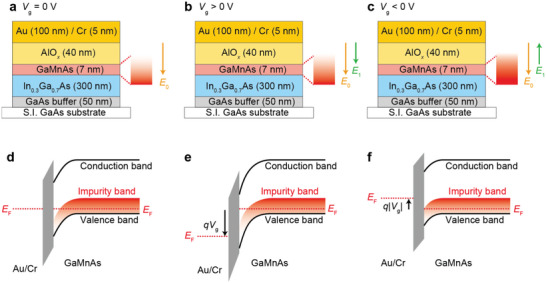
a–c) Schematic illustration of the gate voltage modulation of the interfacial electric field under a) *V*_g_ = 0 V, b) *V*_g_ > 0 V, and c) *V*_g_ < 0 V. d–f) Schematic illustration of the band profile of Au/Cr/AlO*_x_*/(Ga, Mn)As under d) *V*_g_ = 0 V, e) *V*_g_ > 0 V, and f) *V*_g_ < 0 V.

The physical mechanism of the electric field modulation of the SOT magnetization switching in the (Ga, Mn)As layer can be attributed to the successful modulation of the interfacial electric field *E*. As shown in **Figure** **4**a, when *V*_g_ is 0, an internal electric field *E*_0_ is generated because of the existence of a thin depletion layer in the (Ga_0.94_, Mn_0.06_)As layer in the vicinity of the AlO*_x_* layer, as shown in Figure 4d. By applying a positive *V*_g_, the depletion is enhanced, as shown in Figure 4e, which generates an additional interfacial electric field *E*_1_ that points in the same direction as *E*_0_, as shown in Figure 4b. Therefore, the total *E* becomes larger and contributes to a strong spin–orbit interaction, which enhances *H*_R_ and *H*_D_. Then, *H*_R_ and *H*_D_ induce a large spin component along the in‐plane direction, which exerts a strong SOT on the magnetization and enables a highly efficient switching process with a small *J*_c_. When *V*_g_ is negative, holes accumulate near the interface between the AlO*_x_* and (Ga, Mn)As layers based on a capacitor model, which increases the hole concentration near the AlO*_x_* layer, as shown in Figure 4f. Then, the direction of *E*_1_ is reversed, as shown in Figure 4c, and the total *E* is suppressed. The suppressed *E* weakens *H*_R_ and *H*_D_, resulting in a decrease in the strength of the SOT. Therefore, the magnetization switching process is suppressed, and *J*_c_ increases. In addition, it should be noticed that the Oersted field is demonstrated to be negligibly small in the 7 nm thick (Ga_0.94_, Mn_0.06_)As single layer.^[^ ^16^ ^]^

**Figure 5:**
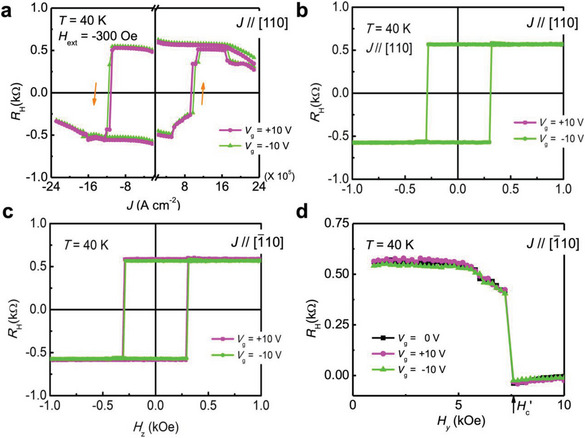
a) Field‐assisted SOT switching with *V*_g_ of 0 and ±10 V in a 7 nm thick (Ga_0.94_, Mn_0.06_)As thin film at 40 K. Here, *J* // [110] and *H*_ext_ = −300 Oe. b,c) Out‐of‐plane magnetic field *H_z_* dependence of the Hall resistance *R*_H_ of (Ga_0.94_, Mn_0.06_)As with *V*_g_ of ±10 V when b) *J* // [110] and c) *J* // [$\bar{1}10$]. d) In‐plane magnetic field *H_y_* dependence of the Hall resistance *R*_H_ of (Ga_0.94_, Mn_0.06_)As with *V*_g_ of 0 and ±10 V. All the measurements were conducted at 40 K.

By changing the direction of *J* from [110] to [110], the *J*_c_ modulation ratio obtained by applying *V*_g_ decreases from 14.5% to 4.9% [=(10.8 − 10.3)/10.3 × 100%], as shown in **Figure** **5**a, where *J*_c_ is estimated to be 10.3 × 10^5^ A cm^−2^ at *V*_g_ = +10 V and 10.8 × 10^5^ A cm^−2^ at *V*_g_ = −10 V. The decrease in the *J*_c_ modulation ratio indicates that both *H*_D_ and *H*_R_ are influenced by *V*_g_ and that the total manipulation effect is much stronger for *J* // [110]. This is reasonable because *H*_D_ and *H*_R_ point in the same direction for *J* // [110], and the modulation effects are superimposed. In contrast, when applying *J* along the [110] axis, the directions of *H*_D_ and *H*_R_ are opposite, which reduces the modulation effect obtained by applying the gate electric field because the total enhancement or suppression effects of *H*_D_ and *H*_R_ cancel out. Hence, a small modulation ratio of *J*_c_ is realized.

To check the influence of the modulation of *H*_c_ by the gate bias voltage, *R*_H_ is measured by sweeping *H_z_* along the [001] direction at *V*_g_ = ±10 V. As shown in Figure 5b,c, with changing *V*_g_, *H*_c_ is nearly constant at approximately 300 Oe regardless of whether the current is applied along the [$\bar{1}10$] or [110] direction, indicating that *H*_c_ is not a determining factor in the electric field control of the SOT switching. In addition, Figure 5c shows that *R*_H_ varies between ±0.587 kΩ when sweeping *H_z_* at *V*_g_ = +10 V. At *V*_g_ = −10 V, *R*_H_ varies between ±0.571 kΩ. Therefore, *R*_H_ can be slightly modulated by *V*_g_ with a modulation ratio of only 2.8% [=(0.587 − 0.571)/0.571 ×100%)]. Additionally, from the results shown in Figure 3a, *R* is estimated to be 193 kΩ at *V*_g_ = +10 V and 186 kΩ at *V*_g_ = −10 V. In ferromagnets, *R*_H_ = *R*_S_ *M* + *R*_0_ *B*, where *R*_S_ is the anomalous Hall coefficient and is proportional to either *R* or *R* ^2^, depending on the AHE mechanism (skew or side‐jump scattering).^[^ ^27^ ^]^ *M* is the magnetization, *R*_0_ is the ordinary Hall coefficient, and *B* is the magnetic field. If *R*_S_ is proportional to *R*, then the saturation magnetization can be considered to be slightly modulated by *V*_g_ with a modulation ratio of only −0.9% [=(0.587/193 − 0.571/186) / (0.571/186) ×100%)]. If *R*_S_ is proportional to *R* ^2^, then the modulation ratio of the saturation magnetization is −4.5% [=(0.587/193^2^ − 0.571/186^2^) / (0.571/186^2^) ×100%)]. However, both values are opposite to *J*_c_ and much smaller than the modulation of *J*_c_ (≈+14.5% for *J* // [110]). Therefore, the saturation magnetization also does not have a significant influence on the electric field control of the SOT switching process. To check the effect of the gate modulation of *H*_an_, we changed the direction of the external magnetic field from [001] to [110] (along the *y* axis), where *H_y_* represents the magnetic field intensity (Figure 5c). From the comparison of *H*_c_ in Figure 5b (*H*_c_ = 300 Oe) and Figure 5c (coercivity *H*_c_′ = 7580 Oe), a 2.3° [=arcsin (300/7580)] misalignment of the magnetic field occurs due to the misalignment of the magnet and/or the sample, resulting in an additional AHE signal. Therefore, an out‐of‐plane component of the external magnetic field with a value of 12 Oe (=300 × sin(2.3°)) exists during the current‐induced SOT switching, but it is negligibly small. Here, the three curves shown in Figure 5c can be found to overlap with each other, which means that *H*_an_ is constant when changing *V*_g_ and that the influence of the modulation of *H*_an_ can also be excluded as a determining factor in the successful manipulation of the SOT switching via the electric field.

## 5 Conclusions

In this work, the manipulation of SOT magnetization switching via a gate electric field is achieved, which can be ascribed to the successful modulation of the interfacial electric field. By applying a positive *V*_g_, the strength of the electric field is enhanced, which strengthens the total effective field in (Ga, Mn)As and assists the magnetization switching. In contrast, a negative *V*_g_ results in a smaller effective field, which decreases the switching efficiency and increases *J*_c_. This finding provides an approach toward reversible modulation of the SOT magnetization switching in a single layer of a spin–orbit ferromagnet via electric fields, which will advance the development of energy‐efficient gate‐controlled spin‐torque devices and help further the understanding of the switching mechanism.

## 6 Experimental Section

### Sample Preparation

A 7 nm thick (Ga_0.94_, Mn_0.06_)As thin film was grown on a semi‐insulating GaAs (001) substrate in an ultrahigh‐vacuum MBE system. After removal of the surface oxide layer of the GaAs substrate at 580 °C, a 50 nm thick GaAs buffer layer was grown to obtain an atomically smooth surface. After that, the substrate was cooled to 450 °C, and a 500 nm thick In_0.3_Ga_0.7_As layer was grown to induce a tensile strain in the (Ga_0.94_, Mn_0.06_)As layer, giving rise to PMA. Then, the sample was cooled to approximately 290 °C for growth of the 7 nm thick (Ga_0.94_, Mn_0.06_)As layer. The growth process was monitored in situ by means of reflection high‐energy electron diffraction. The Curie temperature *T*_C_ of the (Ga_0.94_, Mn_0.06_)As thin film was estimated to be 75 K. After the device fabrication process, the *T*_C_ increased to 112 K, which might be caused by the annealing process resulting from the increase in the sample temperature during the device fabrication process (see Figure S2 in the Supporting Information).

### Transport Measurements

For the SOT measurements, a Keithley 2636A was used as the current source for applying a direct current, and a Keithley 2400 was used for applying a gate voltage. The Hall voltage was measured with another Keithley 2400. The measurements were carried out at 40 K.

## Conflict of Interest

The authors declare no conflict of interest.

## Supporting information

Supporting Information

## Data Availability

The data that support the findings of this study are available from the corresponding author upon reasonable request.
