# Supplementary material for: Electric Field Control of Spin–Orbit Torque Magnetization Switching in a Spin–Orbit Ferromagnet Single Layer
Source: Adv Sci (Weinh). 2023 Jun 17;10(24):2301540. doi: 10.1002/advs.202301540 (PMC10460875; doi:10.1002/advs.202301540)
Supplement: Supplementary file 1 — Supporting Information [file ADVS-10-2301540-s001.pdf]

ADVANCED SCIENCE
Open Access

Supporting Information

for *Adv. Sci.*, DOI 10.1002/advs.202301540

Electric Field Control of Spin–Orbit Torque Magnetization Switching in a Spin–Orbit Ferromagnet Single Layer

Miao Jiang, Hirokatsu Asahara, Shinobu Ohya* and Masaaki Tanaka**

Supporting Information

Electric Field Control of Spin-Orbit Torque Magnetization Switching in a Spin-Orbit Ferromagnet Single Layer

Miao Jiang, Hirokatsu Asahara, Shinobu Ohya, * Masaaki Tanaka**

The crossbar with a size of 0.5 μm (width) × 2 μm (length) was patterned by electron beam (EB) lithography in this work and the device fabrication process is described as follows:

a) Deposition of the Au/Cr electrodes (Figure S1a,b). Firstly, the as-grown sample consisting of $(Ga_{0.94},Mn_{0.06})As$ (7 nm) / $In_{0.3}Ga_{0.7}As$ (500 nm) / GaAs was cleaned with isopropanol (IPA) using an ultrasonic vibrator. The cleaned sample is shown in Figure S1a. After that, the electrodes were patterned by EB lithography. Before the exposure, the sample was coated by a 400-nm-thick positive resist ZEP-520A with a spin-coating rotational speed of 4000 rpm for 60 seconds and then pre-baked at 180 °C for 5 minutes. During the exposure, the dose amount was set to be 105 μC cm^{-2}. After the exposure, a development process was conducted using ZED-N50 for 60 seconds to obtain the pattern for the deposition of the metal electrodes. Here, the Au (100 nm)/ Cr (5 nm) was deposited as the electrodes by EB evaporation, as shown in Figure S1b.

b) Etching to form the crossbar structure (Figure S1c). The crossbar structure was patterned by EB lithography by using a positive resist ZEP-520A-7. The thickness of the resist ZEP-520A-7 is less than 200 nm, thinner than that of the resist ZEP-520A used in step a). The rotational speed during spin-coating was 4000 rpm and the rotational time was 60 seconds. During the exposure process, the dose amount was set to be 60 μC cm^{-2}. Then the exposed sample was developed with the same process as shown in step a) and etched by Ar milling for obtaining a structure, as shown in Figure S1c.

c) Deposition of AlO_x (Figure S1d). As shown in Figure S1d, a 40-nm-thick AlO_x layer was deposited by atomic layer deposition (ALD) as a gate dielectric layer for the application of the electric field. The deposition temperature was set to be 150 °C.

d) Deposition of the gate electrode (Figure S1e). The gate electrode was patterned by using the EB lithography with the same parameters as described in step a). During the exposure process, to

prevent the charging-up effect and resist evaporation due to the heat, the electron beams were scanned twice in a Field Path mode. Then the Au (100 nm)/ Cr (5 nm) metal layers was deposited as the gate electrode by EB evaporation, as shown in Figure S1e.

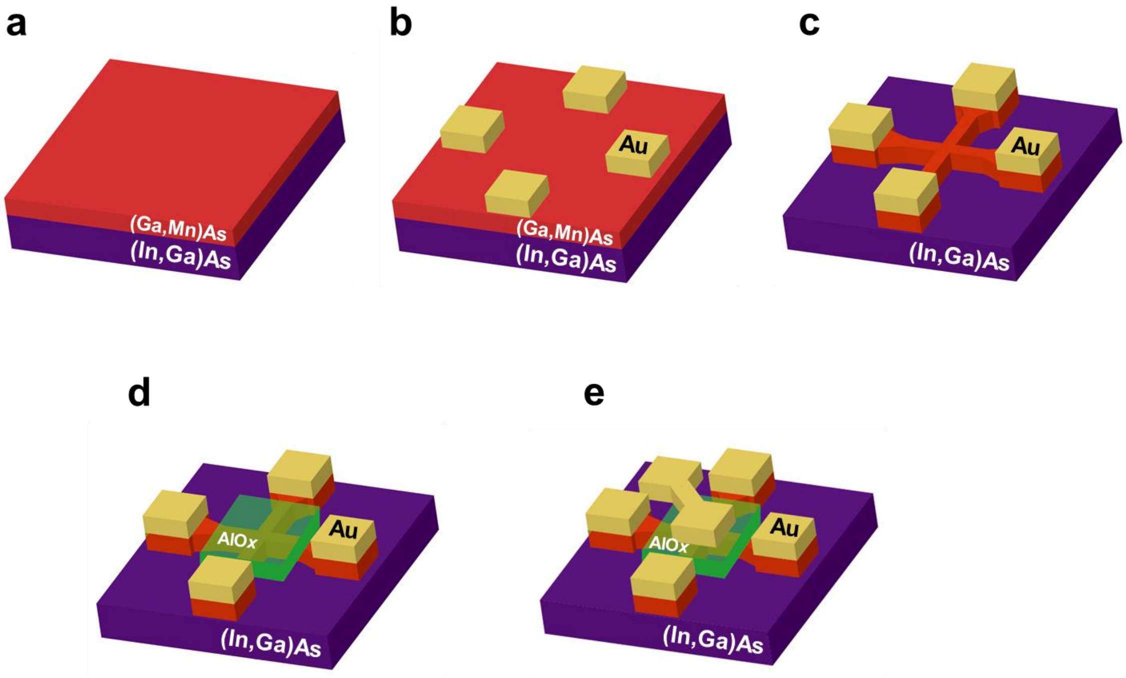

Figure S1 a) As-grown 7-nm-thick $(Ga_{0.94},Mn_{0.06})As$ thin film. b) Deposition of the Au (100 nm)/ Cr (5 nm) layers as the metal electrodes. c) Etching to form the crossbar with a width of 0.5 μm and a length of 2 μm. d) Deposition of AlO_x as a gate insulator by ALD. e) Deposition of the Au (100 nm)/ Cr (5 nm) layers as the gate electrode.

To estimate the Curie temperature T_C, the temperature T dependence of the saturated magnetization M in the 7-nm-thick ($Ga_{0.94}$,$Mn_{0.06}$)As thin film is obtained as shown in Figure S2a. From the results, the M becomes to be around 0 when the T increases to 75 K, which indicates that the T_C of the 7-nm-thick (Ga,Mn)As thin film is 75 K. Figure S2b shows the T dependence of the longitudinal resistance R in the (Ga,Mn)As crossbar with a width of 0.5 μm and a length of 2 μm. When the T decreases from the room temperature to 112 K, the R increases, which results from the decrease in the hole concentration with the decrease of the T. When $T <$ 112 K, as T decreases, the R decreases, which is because the (Ga,Mn)As shows the ferromagnetic property. The spin-flip scattering and the single magnon scattering arising from the ferromagnetically correlated localized Mn spins make the R decrease with decreasing T. From this result, the T_C of the crossbar can be confirmed to be around 112 K, which is higher than that (75 K) measured in the (Ga,Mn)As thin film. This is probably caused by the annealing process resulting from the increase of the sample temeprature during the device fabrication process.

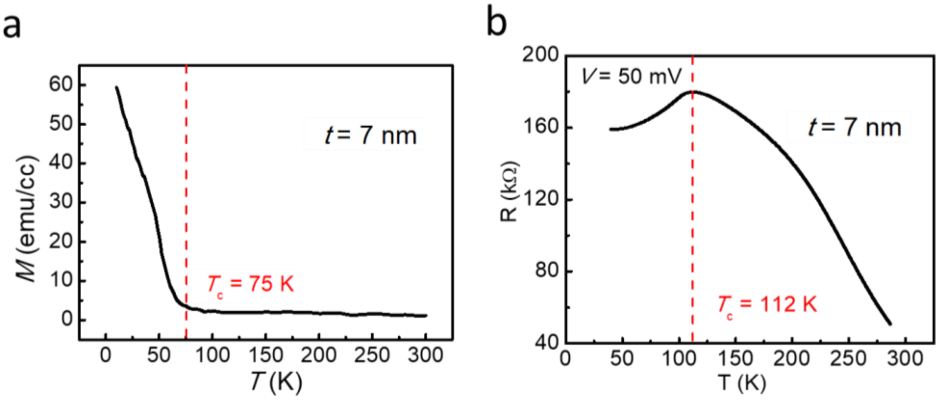

Figure S2. a),b) T dependence of the (a) saturated magnetization M in the 7-nm-thick ($Ga_{0.94}$,$Mn_{0.06}$)As thin film and (b) longitudinal resistance R in the (Ga,Mn)As crossbar device.

Figure S3 shows the manipulation of the SOT switching (J // [$\bar{1}$10]) at different gate voltages V_g of ±5 V, ±10 V, ±15 V and ±20 V at 40 K, from which it can be concluded that the positive V_g makes the J_c decrease and the negative V_g makes J_c increase. This indicates that the magnetization switching behavior is solidly manipulated via pure electronic fields reversibly.

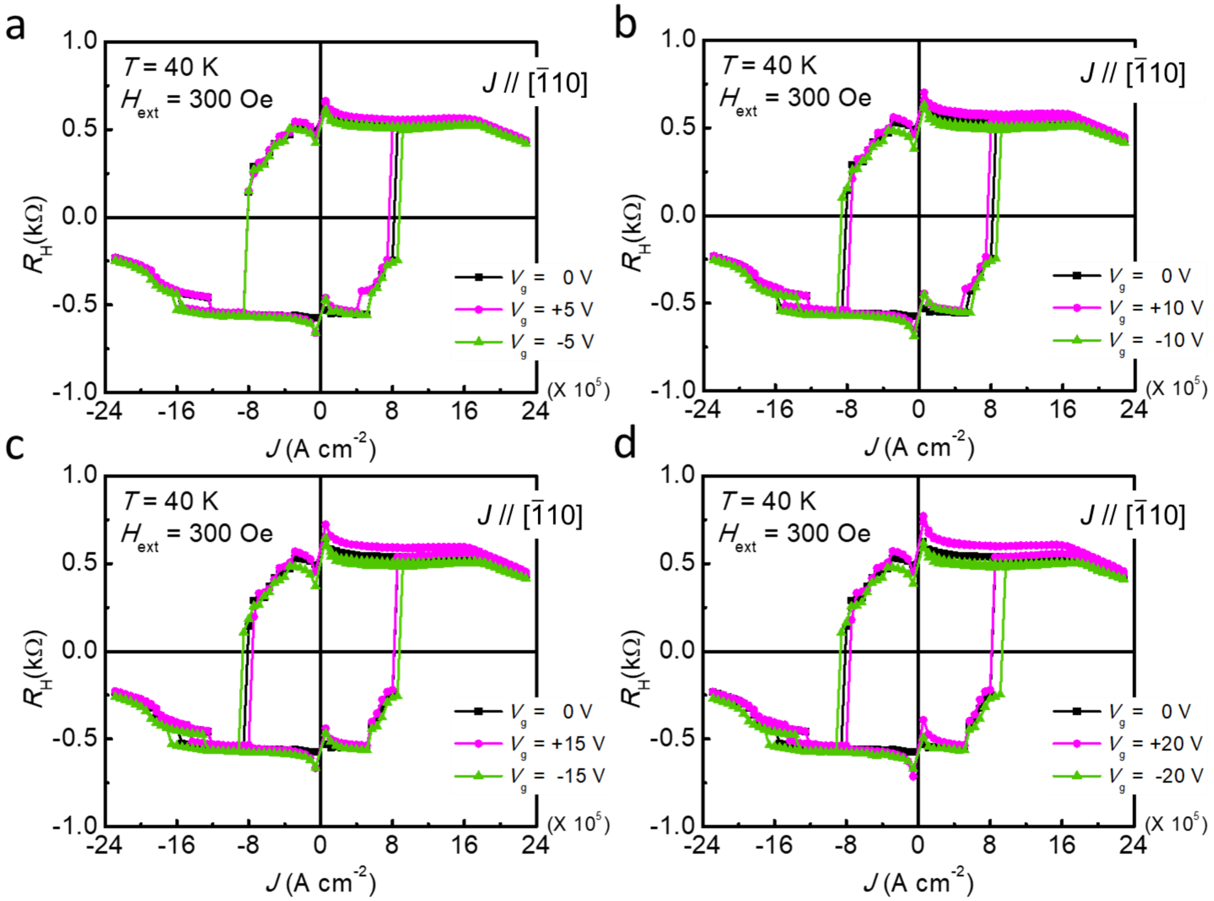

Figure S3. a),b),c),d) Field-assisted SOT magnetization switching with applying the V_g of 0, ±5 V, ±10 V, ±15 V and ±20 V in a 7-nm-thick $(Ga_{0.94},Mn_{0.06})As$ thin film at 40 K. Here, J // [$\bar{1}$10] and H_{ext} = 300 Oe.
